# EV products obtained from iPSC-derived MSCs show batch-to-batch variations in their ability to modulate allogeneic immune responses *in vitro*

**DOI:** 10.3389/fcell.2023.1282860

**Published:** 2023-10-30

**Authors:** Tobias Tertel, Robin Dittrich, Pierre Arsène, Arne Jensen, Bernd Giebel

**Affiliations:** ^1^ Institute for Transfusion Medicine, University Hospital Essen, University of Duisburg-Essen, Essen, Germany; ^2^ Exosla Limited, Cambridge, United Kingdom; ^3^ Brain Repair UG Campus Clinic, Gynaecology, Ruhr University Bochum, Bochum, Germany

**Keywords:** exosomes, EV products, iPSC-derived MSCs, heterogeneity, immune modulation, quality control

## Abstract

Mesenchymal stromal cells (MSCs) have demonstrated therapeutic potential in diverse clinical settings, largely due to their ability to produce extracellular vesicles (EVs). These EVs play a pivotal role in modulating immune responses, transforming pro-inflammatory cues into regulatory signals that foster a pro-regenerative milieu. Our previous studies identified the variability in the immunomodulatory effects of EVs sourced from primary human bone marrow MSCs as a consistent challenge. Given the limited proliferation of primary MSCs, protocols were advanced to derive MSCs from GMP-compliant induced pluripotent stem cells (iPSCs), producing iPSC-derived MSCs (iMSCs) that satisfied rigorous MSC criteria and exhibited enhanced expansion potential. Intriguingly, even though obtained iMSCs contained the potential to release immunomodulatory active EVs, the iMSC-EV products displayed batch-to-batch functional inconsistencies, mirroring those from bone marrow counterparts. We also discerned variances in EV-specific protein profiles among independent iMSC-EV preparations. Our results underscore that while iMSCs present an expansive growth advantage, they do not overcome the persistent challenge of functional variability of resulting MSC-EV products. Once more, our findings accentuate the crucial need for batch-to-batch functional testing, ensuring discrimination of effective and ineffective MSC-EV products for considered downstream applications.

## Introduction

Despite significant progress in science, the human society is still suffering from many diseases without having real cure options. Among them are many diseases whose symptomologies are closely associated with proinflammatory processes, ranging from early neonates, e.g., in hypoxia induced encephalopathy patients, to elder individuals suffering from a variety of different degenerative diseases or ischemia reperfusion injuries, including ischemic stroke and myocardial infarction. Proposing that symptoms of many of such diseases can be improved by cell replacement therapies, big hopes were attributed to non-hematopoietic stem cell research. Aside embryonic stem cells and later to induced pluripotent stem cells (iPSCs), many researches focused on the therapeutic potential of mesenchymal stromal cells (MSCs), whose differentiation capabilities were initially considered to be far above the mesenchymal linage (Munoz-Elias et al., 2003; Pittenger and Martin, 2004). Lacking any teratogenic features, MSCs quickly were discussed as a promising *of-the-shelf* product for many acute degenerative diseases including ischemic stroke and myocardial infarction. Consequently, their interaction with allogenic immune cells were studied in detail. Starting with publications in 2002, it turned out that MSCs supress proinflammatory and promote regulatory functions of the immune system (Bartholomew et al., 2002a; b; Di Nicola et al., 2002; Meisel et al., 2004). Meanwhile, coupled to their safety, MSCs raised from various tissue sources have been applied in more than 1,500 NIH registered clinical trials to various patient cohorts, either with the intention to use them as cell replacement or immunomodulatory agent (clinicaltrials.gov). Indeed, positive outcomes were reported in many but not all clinical studies. For example, a phase III clinical trial failed to show efficacy in a steroid-refractory acute Graft-versus-Host Disease (aGvHD) patient cohort (Galipeau, 2013; Kebriaei et al., 2020).

Over the years it became obvious that despite some shared features (Dominici et al., 2006), MSCs provide a heterogenous cell entity with tissue specific and inter-individual differences (Phinney et al., 1999; Vogel et al., 2003; Phinney, 2012; Radtke et al., 2016). Apparently, not all MSC types mediate therapeutic effects. Notably, MSC researchers have just started to discuss the relation of the MSC heterogeneity on their clinical potentials more intensively (Dunn et al., 2021; Galipeau et al., 2021). In terms of their mechanism of action, it turned out that MSCs mainly mediate their beneficial effects via their secretome and not by cell replacement or direct molecular interactions (Gnecchi et al., 2005; Gnecchi et al., 2006; Timmers et al., 2007; Timmers et al., 2011). Upon searching for the active components in the MSCs’ secretome, therapeutic activities were recovered in fractions being highly enriched for extracellular vesicles (EVs) including exosomes and microvesicles (Bruno et al., 2009; Lai et al., 2010). As non-self-replication units with sizes below 200 nm, EV products provide several principal advantages over cellular products, e.g., EVs lack endogenous tumour formation potentials and resulting products can be sterilized by filtration (Lener et al., 2015). According to their advantages over cells, MSC-EV products are increasingly considered as therapeutic agents (Borger et al., 2020b; Gimona et al., 2021). Prepared in accurate manner, MSC-EV products were shown to exert the same therapeutic effects than their parental cells (Bruno et al., 2009; He et al., 2012; Doeppner et al., 2015). Even though some successful MSC-EV treatments have been reported in patients (Kordelas et al., 2014; Nassar et al., 2016; Kwon et al., 2020; Sengupta et al., 2020; Warnecke et al., 2021; Lightner et al., 2023), studies on different pre-clinical disease models, ranging from ischemic stroke to GvHD, revealed comparable to MSC products, not all MSC-EV products contain all critical activities required for improving respective disease symptoms (Wang et al., 2020; Van Hoecke et al., 2021; Madel et al., 2023). Thus, for effectively translating MSC-EVs into the clinic, two different limitations of current MSC-EV production strategies need to be solved. On the one hand, the heterogeneity of MSC-EV products need to be addressed by defining appropriate quality control criteria at the metric and functional level (Witwer et al., 2019; Gimona et al., 2021; Papait et al., 2022), on the other hand concepts for the scaled MSC-EV production need to be established (Grangier et al., 2021; Staubach et al., 2021). To this end, even upon applying the best production protocols, aging processes finally resulting in replicative senescence of the EV-releasing MSCs will limit the scalability of the production process. Primary MSCs raised from adult donor materials typically exhibit restricted expansion capabilities, consequently limiting the size of manufacturable MSC batches. Considering comparable dosing strategies for MSC-EV products than for MSCs, i.e., at least EVs harvested from the supernatant of 10^6^ MSCs per kg body weight (Kordelas et al., 2014), also respective EV products can only be manufactured in moderate batch sizes. Thus, it is an important goal to define strategies warranting higher expansion rates of the EV-releasing MSCs. Since MSCs derived from iPSCs typically achieve more population doublings *in vitro* than MSCs from adult donor materials (Lian et al., 2010), iPSC-MSCs (iMSCs) appear as an attractive cell source for the production of therapeutic EVs. Consequently, to test whether iMSCs may allow the manufacturing of potent EV products, we have set up and compared different protocols to raise iMSCs form clinical-grade iPSCs. Resulting iMSCs were expanded and phenotypically characterized after different passages. Furthermore, EVs were prepared from their conditioned culture media and obtained iMSC-EV products characterized at the phenotypic as well as at the functional the level.

## Material and methods

### Induction of the development of iPSC towards MSCs

iPSCs used for MSC derivation were initially derived from cord blood CD34^+^ cells under GMP-compliant conditions in a clean room setting and using full environmental monitoring and documentation according to international GMP standards. Detailed procedures and characterization of these cells will be published elsewhere. Briefly, CD34^+^ cells were reprogrammed using a cocktail of episomal vectors following a methodology similar to published procedures (Okita et al., 2013). Resulting clonal iPSC lines showed normal karyotypes and no acquired mutations in a defined set of cancer-causing genes. Their differentiation potential was validated using various assays including protocols for the induction of endothelial, cardiac, neural and immune cells. An iPSC line fulfilling all specified criteria was selected and employed under R&D conditions in the present study.

Prior to MSC development induction, iPSCs were maintained in iPS Brew XF medium (Miltenyi Biotec, Bergisch Gladbach, Germany) using enzymatic passaging with Accutase (Sigma-Aldrich, Taufkirchen, Germany) in the presence of 10 µM Y-27632 (R&D Systems, Minneapolis, United States). iPSCs were propagated on 6-well tissue culture plates coated with iMatrix-511 (Amsbio, Abingdon, United Kingdom; available in GMP quality: 3 µL per well in 2 mL of culture medium containing Y-27632 for 1 h at 37°C). A new protocol was devised for inducing MSC development by stimulation of the cells with a single compound, the WNT activator CHIR99021 (STEMCELL Technologies, Vancouver, Canada): iPSCs were reseeded onto laminin-coated plates at 3 × 10^3^/cm^2^ in iPSC culture medium containing Y-27632. The next day (day 0), medium was replaced with 4 mL of MSC induction medium (iPS Brew XF + 4 μM CHIR99021). From d3 onwards, medium was replaced with 3 mL of fresh MSC induction medium every day for another 3 days. On d6, cultures were switched to MSC medium. Partially detaching clusters of cells were kept in the same well at this time point. MSC-like cells tended to grow out from the higher-density attached clusters over the next days. After ∼1 week, when cultures reached full confluence, cells were dissociated by enzymatic digestion using Accutase (anticipated to be available in GMP quality) and replated at 1:2 in MSC medium, again on iMatrix. These passages were termed p1. A 1:2 split ratio was kept for another 3 passages, albeit without further coating the tissue culture dishes. From P4 onwards, the emerging iPSC-MSCs showed a homogeneous morphology and upon reaching ∼90% confluence were split revealing yields of ∼200,000 cells per well of 6-well plates. MSC medium was replaced every other day (2 mL, or 3, 4 mL over weekends). Within the E8 experimental series, MSC medium was composed of DMEM/F12 base medium (Thermo Fisher Scientific, Darmstadt, Germany) with 10% human platelet lysate (PL Biosciences, Aachen, Germany, available in GMP quality) and penicillin/streptomycin supplemented to low-glucose DMEM (Thermo Fisher Scientific). Within the E13 and E19 experimental series StemMACS MSC expansion medium XF (Miltenyi Biotec) was used as MSC medium and hPL-based medium for the expansion of obtained iMSCs.

### Expansion of iMSCs and preservation of conditioned media (CM)

Passage 10 aliquots of E8, E13 and E19 iMSCs, all produced at Catalent, were shipped on dry ice to the University Hospital Essen. For expansion, 400,000 iMSCs were seeded in a Nunc Triple Flask (500 cm^2^ surface area) in 100 mL DMEM low (PAN-Biotech, Aidenbach, Germany) supplemented with 10% human platelet lysate (hPL; Batch 2/20 II AG Giebel), 1% Penicillin/Streptomycin/Glutamin (Thermo Fisher Scientific) and 0.1% Heparin (Ratiopharm, Ulm, Germany).

For the collection of iMSC-CM, following passaging of iMSCs, upon reaching 50% confluence, initial media were exchanged and CM collected after 48 h, a time point, iMSCs already reached 80%–90% confluency. In total, 200 mL of CM (two Nunc Triple Flasks) were collected per passage. The CM were pre-processed by 15 min centrifugation at 2,000 x g and 4°C, and were stored at −20°C until further procession.

### Characterization of iMSCs

Expanded iMSCs were characterized by flow cytometry as described before (CytoFLEX S; Software Cytexpert 2.3; Beckman Coulter, Krefeld, Germany) (Kordelas et al., 2014). For the phenotypic characterization iMSCs were labelled with antibodies directed against *bona fide* and negative MSC marker proteins (positive marker CD44, CD73, CD90 and CD105; negative markers: CD14, CD31, CD34 and HLA-DR) (Dominici et al., 2006). Details on the antibodies are provided in Supplementary Table S1. The obtained data were analysed with Kaluza Analysis 2.1 software (Beckman Coulter).

### Differentiation assays

Adipogenic and osteogenic differentiation capabilities were scored for each independent iMSC line during passages 11 and 19. Comprehensive information about the composition of the osteogenic differentiation medium, adipogenic induction medium, and adipogenic maintenance medium used in these experiments, including specific component quantities, are provided in Supplementary Table S2.

For osteogenic differentiation, cells were seeded at a density of 1 × 10^5^ cells per well in a 12-well plate, using 1 mL of culture medium. Upon reaching 80% confluence, the culture medium was replaced with 1 mL of osteogenic differentiation medium in two of the wells to initiate differentiation, while the third well served as a control. The medium was refreshed every 3, 4 days. After a period of 3 weeks, Alizarin Red staining was performed. The cells were washed with phosphate-buffered saline (PBS; Thermo Fisher Scientific) and fixed using 70% ice-cold ethanol for 10 min. After washing with distilled water, the cells were stained with 300 μL of Alizarin Red (Roth, Karlsruhe, Germany) per well for 10 min. Subsequently, five washing steps with distilled water were carried out. After adding PBS, the stained cells were documented by light microscopy. For subsequent quantification, plates were dried and kept at −20°C.

The adipogenic differentiation procedure mirrored the osteogenic differentiation process. Cells were seeded at a density of 1 × 10^5^ cells per well in a 12-well plate, in 1 mL of culture medium. Once the cells reached 80% confluence, the culture medium was replaced by 1 mL of adipogenic induction medium in two wells, while the third well was maintained as a control. Thereafter, media were exchanged every 3, 4 days; of note, in an alternating manner, adipogenic induction and maintenance media were used. After 3 weeks, the cells were stained with Oil Red-O. Per three wells, a working solution of Oil Red-O was prepared, a mixture of 3.3 mL Oil Red-O stock solution (150 mg/50 mL isopropanol, Sigma-Aldrich) and 1.7 mL of distilled water that was cleared by filtration after 10 min and was used within 3 hours. The cells were then washed with PBS and fixed with 4% paraformaldehyde (Roth) for 2 min at −20°C. After washing with 50% ethanol, the cells were stained with 300 μL of the Oil Red-O working solution per well for 10 min. Subsequently they were washed, ones with 50% ethanol and five times with distilled water. After refilling each well with PBS, the cells were documented by light microscopy.

### Preparation of iMSC-EVs

For the EV preparation cryopreserved CM were thawed at 4°C. For preparation of the EVs, thawed CM of p11 to p13, p14 to p16 and p17 to p19 were pooled for each of the 3 independent experimental series, E8, E13 and E19. Thereafter, EVs were prepared according to an established standard procedure, i.e., via polyethylene glycol precipitation followed by ultracentrifugation (PEG-UC) exactly as described previously (Kordelas et al., 2014; Ludwig et al., 2018; Borger et al., 2020a). Obtained EV pellets from the CM of 4 × 10^7^ MSC equivalents were solved in 1 mL 10 mM HEPES in 0.9% NaCl buffer.

### Nanoparticle tracking analysis (NTA)

Nanoparticle tracking analysis (NTA) was performed to determine the particle concentration and average size distribution of obtained iMSC-preparations (Dragovic et al., 2011; Sokolova et al., 2011). The analyses were conducted on a ZetaView PMX-120 platform, equipped with the software version 8.03.08.02 (ParticleMetrix, Meerbusch, Germany).

Briefly, given EV samples were initially diluted in sodium chloride (NaCl; B. Braun, Melsungen, Germany) solution. For analysis, 1 mL of the diluted sample was loaded into the flow cell of the ZetaView for and recorded for 55 s at all 11 detection positions. Obtained data were compiled and analysed, with the mean of the results serving as the reported value for the concentration and the average size of particles per sample. NTA analyses were conducted using a setting allowing the detection of particles with sizes from 5 to 200 nm: Positions: 11; Cycles: 5; Quality: medium; Minimum brightness: 20; Minimum particle size: 5; Maximum particle size: 200; Trace length: 15; Sensitivity: 75; Shutter speed: 75; Frame rate: 30.

### Bicinchoninic acid assay (BCA)

In this study, a BCA kit (Thermo Fisher Scientific) was used for assessing the protein concentration. All analyses were performed in duplicates. Briefly, an array of bovine serum albumin (BSA) standards was established through a series of dilutions in 0.9% NaCl. These dilutions ranged from a concentration of 2000 μg/mL (from a stock of 300 μL BSA and no NaCl) down to zero. Simultaneously, a working standard (WS) was prepared for a microplate assay. The WS was constituted at a ratio of 1:50, involving 1 part of kit included Reagent B to 50 parts of Reagent A. Specifically, for the experiment involving 30 samples, a total volume of 6 mL WS was prepared. Subsequently, samples with protein contents exceeding the maximal detection limit were diluted at the ratios of 1:5 and 1:10. For the 1:5 dilution, 12 μL of given samples were mixed with 48 μL of 0.9% NaCl (B. Braun). For the 1:10 dilution, 6 μL of the sample was combined with 54 μL of 0.9% NaCl (B. Braun). 25 μL of each dilution were applied onto a 96-well microplate (Greiner, Essen, Germany), followed by the addition of 200 μL of the prepared WS to each well. Microplates were subsequently incubated at 37°C for 30 min. Before analysis, microplates were cooled to room temperature. The absorbance was measured in a microplate reader (Biotek PowerWave XS, Berlin, Germany) at 562 nm and analysed with the GenX5 software (Biotek).

### Western blot

Western blot analyses were performed for each individual series of experiments (E8, E13, and E19) as well as for all three independent experiments in parallel to each other as described previously (Ludwig et al., 2018). Briefly, 5 µL of iMSC-EV preparations were solved and denatured using Laemmli sample buffer (4×) with dithiothreitol (AppliChem GmbH, Darmstadt, Germany) and separated by one-dimensional sodium dodecyl sulfate-polyacrylamide gel electrophoresis on 12% gels. Proteins were transferred to polyvinylidene fluoride membranes (Sigma-Aldrich), which were subsequently blocked with Tris-buffered saline and 0.1% Tween 20 containing 5% (w/v) skim milk powder (Sigma-Aldrich). To detect EV marker proteins, the following antibodies were used: anti-Syntenin (clone EPR8102; Abcam, Cambridge, United Kingdom), anti-CD81 (clone JS-81; BD Biosciences, San Jose, CA, United States), anti-CD9 (clone VJ1/20.3.1; kindly provided by Francisco Sánchez, Madrid, Spain), and anti-CD63 (clone H5C6; BioLegend, San Diego, CA, United States). Bound antibodies were counter-labeled with horseradish peroxidase-conjugated secondary antibodies (Dianova, Hamburg, Germany), and signals were recorded after addition of SuperSignal West Femto Maximum Sensitivity Substrate (Thermo Fisher Scientific) on a Fusion FX7 chemiluminescence detection system (Vilber Lourmat Deutschland GmbH, Eberhardzell, Germany). 16-bit images were acquired using image acquisition software (EM-MENU, v4.09.83). Image post-processing was carried out using ImageJ 1.52b software (National Institutes of Health, Bethesda, MD, United States).

### Single EV analysis by imaging flow cytometry

Imaging flow cytometry analyses were performed for each individual series of experiments (E8, E13, and E19) as well as for all three independent experiments in parallel to each other. Unless mentioned otherwise, MSC-EV preparations were stained exactly as described before (Tertel et al., 2020a; Tertel et al., 2020b). Briefly, 5 µL of given iMSC-EV samples were stained with APC-conjugated mouse anti-human CD63 (clone MEM-259; EXBIO Praha, Czech Republic) or, for control experiments, with mouse IgG1 isotype (clone MOPC-21; BioLegend). Additional 5 µL samples of obtained MSC-EV preparations were stained with PE-conjugated mouse anti-human CD9 (clone MEM-61; EXBIO Praha) or FITC-conjugated mouse anti-human CD81 (clone JS-84; Beckman Coulter) antibodies, respectively. Unstained samples and buffer controls without MSC-EVs but with antibodies served as additional controls. All samples were incubated for 1 h in the dark at room temperature, before analysis. Anti-CD9 labelled EV samples were diluted 100-fold and anti-CD63 as well as anti-CD81 stained sample 40-fold with PBS (pH 7.4; Thermo Fisher Scientific).

Without performing any further processing, samples were analysed on an Amnis ImageStreamXMk II instrument (Cytek Bioscience, California, United States). Samples were applied in U-bottom 96-well Falcon plates (Corning GmbH, Kaiserslautern, Germany) and analysed in triplicate with a 5-min acquisition time per well. All data were acquired at ×60 magnification at low flow rate and with the removed beads option deactivated. Additional information is provided in Supplementary Tables S2, 3. Data analysis was performed using IDEAS 6.2 software (Cytek Bioscience) as described previously (Tertel et al., 2020a; Tertel et al., 2020b). The fluorescence intensities of recorded objects were plotted against side scatter values, and the spot counting feature was used to analyse images for coincidences (swarm detection). Multiplets were excluded; all remaining events with low side scatter (<500) and a fluorescent intensity higher than 300 were considered for concentration calculations (Tertel et al., 2020b).

### Multi-donor mixed lymphocyte reaction (mdMLR) assay

The immunomodulatory activities of samples of the obtained iMSC-EV preparations were evaluated individually within the mdMLR assay exactly as described previously (Bremer et al., 2023; Nardi Bauer et al., 2023). Briefly, peripheral blood mononuclear cells (PBMCs) from 12 different donors were isolated by conventional Ficoll density gradient centrifugation and pooled using identical cell numbers from each donor. Aliquots of PBMC pools were stored in the vapor phase of liquid nitrogen until usage. Upon thawing, PBMCs were cultured in RPMI 1640 medium (Thermo Fisher Scientific) supplemented with 10% human AB serum (produced in-house), 100 U/mL penicillin, and 100 μg/mL streptomycin (Thermo Fisher Scientific). A total of 6 × 10^5^ cells in a final volume of 200 µL per well were cultured at 37°C and a 5% CO_2_ atmosphere in the presence or absence of iMSC-EV preparations in 96-well U-bottom Falcon plates (Frima), respectively. For functional testing, 5 µL samples of given iMSC-EV preparations were applied to the respective wells. After 5 days of culture, the cells were harvested and stained with a cocktail of fluorescently labelled antibodies: anti-CD4, anti-CD8, anti-CD25 and anti-CD54 antibodies. Dead cells were identified as 7-aminoactinomycin D (Beckman Coulter) incorporating cells. Data acquisition was performed on a CytoFLEX flow cytometer with CytExpert 2.3 software (Beckman Coulter). The obtained data were analysed with Kaluza Analysis 2.1 software (Beckman Coulter). More details for the antibodies are provided in Supplementary Table S4.

## Statistics

All collected data were at first tested for normal distribution. Subsequently, a two-tailed Mann-Whitney test was performed to compare the means of two independent groups. The numbers presented in the results refer to the *p*-values obtained from these tests. Differences in the coefficient of variation were determined with the Brown-Forsythe test. Data were visualized and tests performed with Prism 8.4.2 software (GraphPad Software, San Diego, CA, United States).

## Results

### iPSCs can create MSC-like cells

Our study aimed to investigate whether GMP-compliant iPSCs can be used to generate iMSCs that fulfil the *bona fide* criteria of primary MSCs and display EVs over several passages with immunomodulatory properties. In a newly established protocol, MSC-like cells were derived in 2D culture based on 6-day lasting WNT stimulation as the only cell signalling perturbation, followed by propagation in MSC-supportive media. In three independent experiments, MSC-like cells were raised from the same iPSC line. In the first experiment (E8), derived MSC-like cells were initially kept in a human platelet lysate-containing (hPL) medium, while in the second and third experiment (E13 and E19) derived MSC-like cells that were finally expanded in the hPL supplemented medium were initially kept in a commercial MSC medium. Independent of the medium of choice, iPSCs were able to generate cells in all three independent experiments fulfilling the morphological phenotypic characteristics of *bona fide* MSCs (data not shown).

### iPSC-derived MSC-like cells can extensively be expanded but do not overcome the Hayflick limit

**FIGURE 1:**
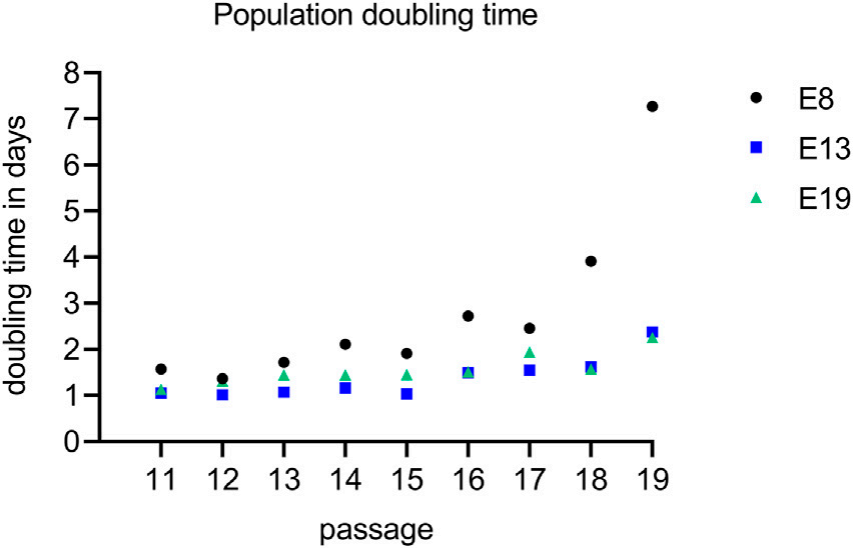
iPSC-derived MSC-like cells became senescent after passage 19. The population doubling rate of iPSC-derived MSCs were recorded in all experimental series between passage 11 (E8: black, E13: blue and E19: green) until they became senescence that was in passage 20. The graph depicts the number of population doublings per days across various passages. To assess the differences between the MSC inducing protocols, a statistical analysis was conducted using the Mann-Whitney test. Specifically, E8 was compared to both E13 and E19, yielding a statistically significant difference with a *p*-value of 0.0078.

The population doubling times of iPSC-derived MSC-like cells were recorded over several passages (Figure 1). None of the cells could be efficiently expanded beyond passage 19. For MSC-like cells obtained in E8, the population doubling time ranged from 1.6 days at passage 11 to 7.3 days at passage 19. Similarly, for those obtained in E13, the population doubling time ranged from 1.0 days at passage 12 to 2.4 days at passage 19 and for those obtained in E19, from 1.1 days at passage 11 to 2.3 days at passage 19. With an average of 3 populations doublings per passage, under the given conditions, the iPSC-derived MSC-like cells were not able to exceed the Hayflick limit.

### Initial culture conditions affect consistency of CD105 expression in iPSC-derived MSC-like cells

**FIGURE 2:**
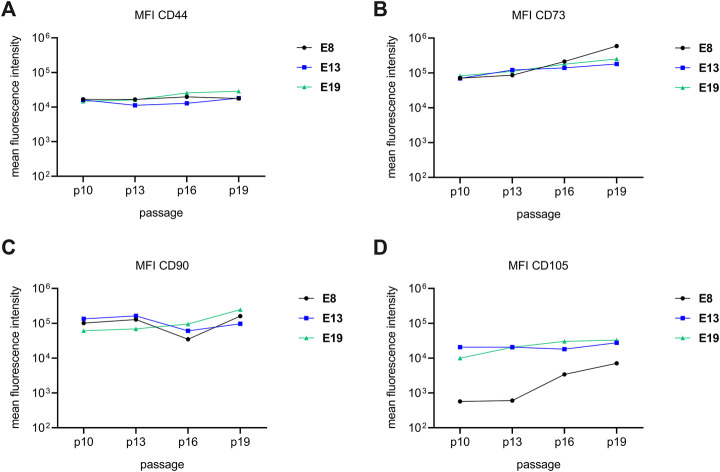
iPSC-derived MSC-like cells reveal cell surface phenotypes of bona fide MSCs. The cell surface marker expression profiles of MSC-like cells obtained in 3 independent experiments (E8: black, E13: blue and E19: green) were evaluated after 4 different passages, passage 10, 13, 16 and 19. Mean fluorescent intensities of anti-CD44 **(A)**, anti-CD73 **(B)**, anti-CD90 **(C)** and anti-CD105 antibody labelled MSC-like cells are depicted for each of the analysed passages. The lines are a visual assistance and do not represent a continuous measurement. The gating strategy and examples of primary data are shown in Supplementary Figure S1.

For exploring the *bona fide* criteria of obtained MSC-like cells, flow cytometry analyses of obtained MSC-like cells were performed after passage 10, 13, 16 and 19. Consistently, all MSC-like cells expressed relative constant levels of the cell surface antigens CD44, CD73 and CD90 over all passages (Figure 2). Furthermore, all cells were negative for CD14, CD31, CD34 and HLA-DR (Supplementary Figure S1). In contrast, we observed inconsistencies in the CD105 expression. While MSC-like cells that were initially raised in the commercial MSC-medium, expressed CD105 in comparable amounts over all evaluated passages, MSC-like cells of E8 that were raised right from the beginning in hPL supplemented media, had a low amount of CD105 in passages 10 and 13 but showed increased levels in passage 16 and 19 (Figure 2). Accordingly, we concluded that the initial culture conditions have long lasting impacts on the phenotypic properties of obtained MSC-like cells.

### iPSC-derived MSC-like cells exhibit capacities to differentiate into adipocytic and osteocytic cells

**FIGURE 3:**
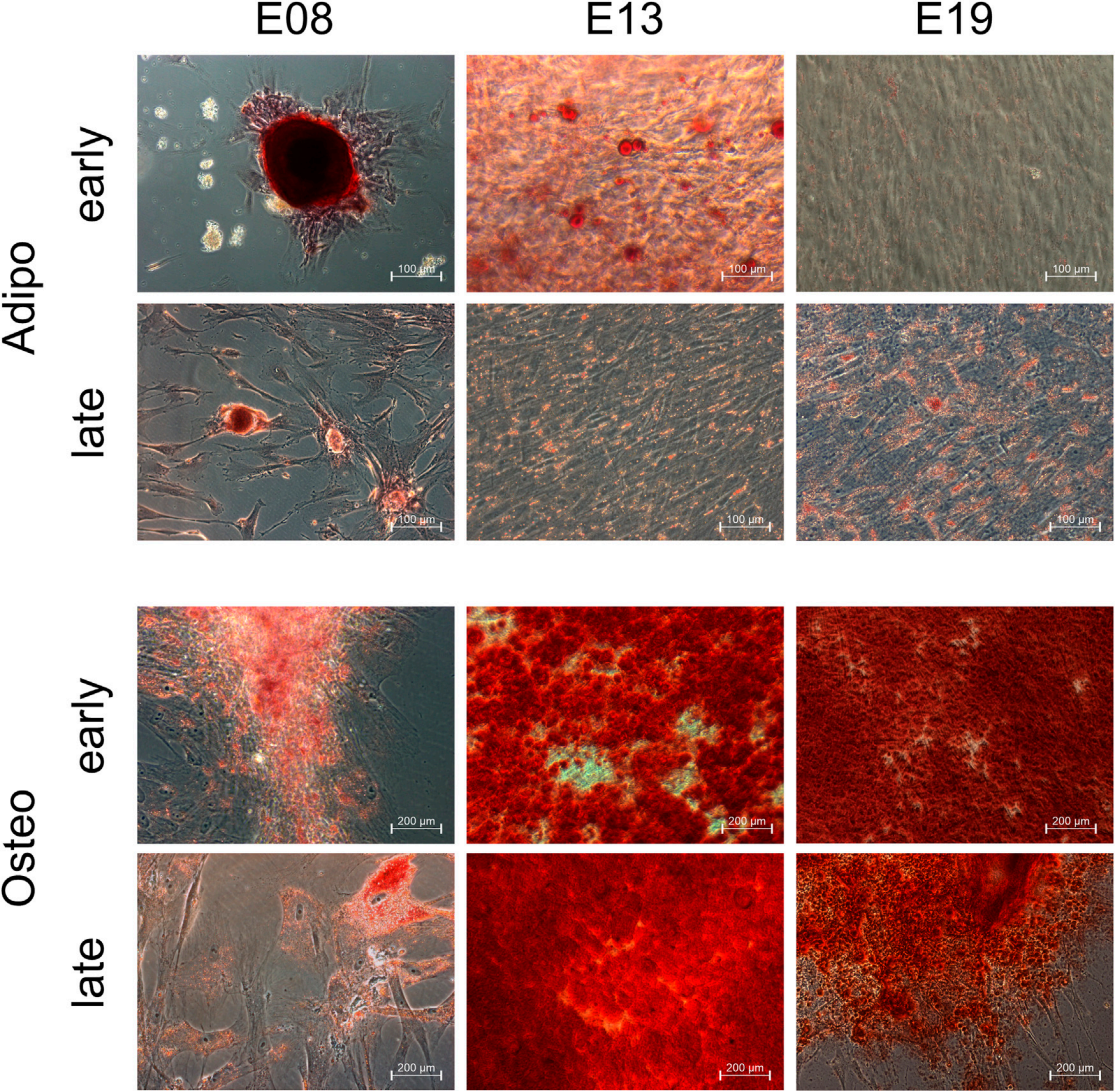
iPSC-derived MSC-like cells can differentiate into adipogenic and osteogenic cells. iPSC-derived MSC-like cells obtained in E9, E13 and E19 were induced to differentiate into adipogenic and osteogenic cells after passage 10 (early) and passage 19 (late).

Experts of the International Society of Extracellular Vesicle (ISEV) recommended to confirm at least one of the trilineage potentials of MSCs raised for the MSC-EV production (Witwer et al., 2019). Here, the iPSC-derived MSC-like cells were checked for their ability to differentiate into adipogenic and osteogenic derivatives at different passages, i.e., after passage 10 and after passage 19. MSC-like cells obtained in E8 formed distinct fat structures, MSC-like cells of the early passage much more pronounced than those from the later passage. MSC-like cells obtained in E13 and E19, also revealed adipogenic differentiation capabilities, even though to a lesser extend (Figure 3). In addition, MSC-like cells of all experiments and all passages were able to differentiate into cells with osteogenic features (Figure 3). Since all MSC-like cells were able to differentiate along the osteogenic linage and revealed adipogenic differentiation potentials, all *bona fide* MSC characteristic which we have tested for and which are recommended to be tested for the EV production are fulfilled. Thus, we concluded that the obtained MSC-like cells are indeed MSCs, for the rest of the manuscript now termed iMSCs.

### iMSCs release *bona fide*-EVs

Next, to learn whether iMSCs reveal comparable EV release characteristics than primary MSCs, iMSC-EVs were prepared from the CM collected during their expansion. To this end, CM of p11-p13, p14-p16 and p17-p19 of the given iMSC lines (E8, E13 and E19) were pooled and EVs prepared with our well-established and standardized PEG-UC method (Kordelas et al., 2014; Ludwig et al., 2018; Borger et al., 2020a). Considering that most particles in given EV preparations correspond to non-vesicular particles and only minor protein contents recovered in EV samples derive from EVs (Droste et al., 2021), for solving obtained EV preparations, we applied our original strategy and diluted the EV-harvest of the CM of 4 × 10^7^ iMSCs to 1 mL (Kordelas et al., 2014).

In the present study, samples of all iMSC-EV preparations were evaluated using nanoparticle tracking analysis (NTA), protein concentration analysis, and imaging flow cytometry (IFCM) for CD9, CD63 and CD81 objects and Western blot for CD9, CD63, CD81 and Syntenin (Figure 5). All data are provided in Supplementary Table S5 and are depicted in Figure 5. For the EV-preparations obtained from E8 iMSC-CM, the NTA demonstrated particle counts per ml ranging from 3.6 × 10^11^ to 5.7 × 10^11^ and average particle sizes of 118.3 nm. The protein concentration measurements varied between 6.3 and 14.1 mg/mL. According to IFCM, CD9, CD63, and CD81 object concentrations per ml ranged from 3.0 × 10⁹ to 8.6 × 10⁹, 5.7 × 10⁸ to 2.2 × 10⁹ and 5.8 × 10⁸ to 2.5 × 10⁹, respectively. In the E13 samples, we observed NTA particle concentrations per ml in the range of 1.8 × 10^11^ to 3.7 × 10^11^ and average particle sizes of 120.8 nm. The protein concentration varied from 3.8 to 7.3 mg/mL. IFCM measurements for CD9, CD63 and CD81 objects per ml varied between 1.8 × 10⁹ and 5.8 × 10⁹, 1.6 × 10⁸ and 2.9 × 10⁸ and 2.4 × 10⁸ and 9.3 × 10⁸, respectively. Regarding the E19 samples, the NTA analyses showed particle concentrations per ml from 2.6 × 10^11^ to 4.0 × 10^11^ and average particle sizes of 110.7 nm. The protein concentrations ranged from of 6.9–8.3 mg/mL. IFCM results for CD9, CD63 and CD81 objects per ml were within the range of 2.4 × 10⁸ to 5.6 × 10⁸, 9.5 × 10⁷ to 2.4 × 10⁸, and 1.0 × 10⁸ to 2.2 × 10⁸, respectively. The gating strategy with example plots is depicted in Supplementary Figure S2.

**FIGURE 4:**
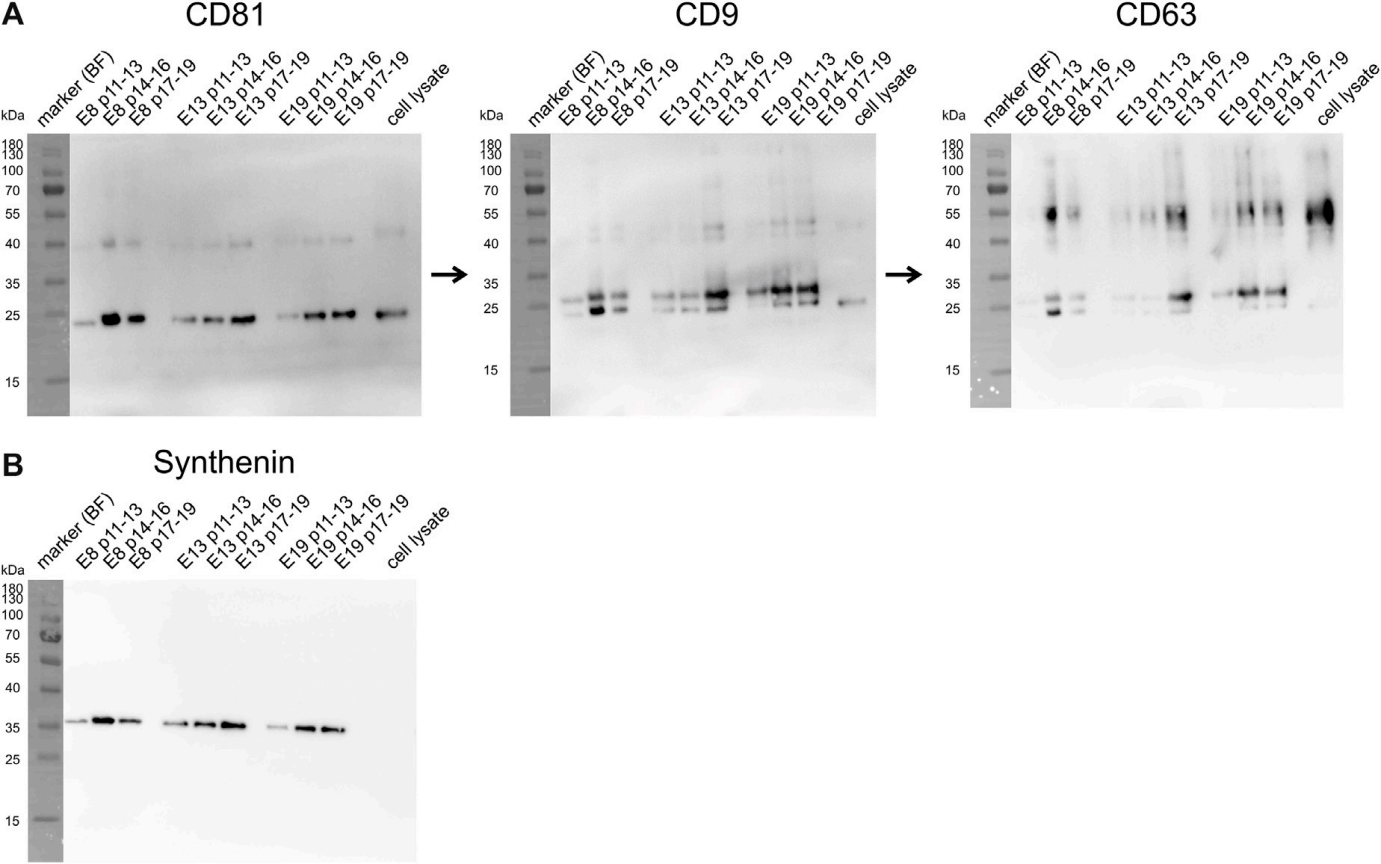
The obtained iMSC-EV preparations contained varying concentrations of the EV marker proteins CD81, CD9, CD63 and Syntenin. **(A)** This figure presents the results of a sequential Western blot analysis targeting the proteins CD81, CD9, CD63 and Syntenin. Each of the tetraspanins was successively probed on the same membrane, starting with CD81, then CD9, and finally CD63. Syntenin was detected on a separate blot **(B)**. The proteins were initially separated by size via SDS-PAGE and subsequently transferred onto a membrane. Their presence and approximate size were revealed using chemiluminescence detection. The marker bands were documented under bright-field illumination (BF), all others according to their chemiluminescence. Images of the bright field bands were superimposed onto the chemiluminescence blot image, explaining the contrast between the protein and marker band backgrounds. Marker bands were labelled with their representing molecular weights in kilodaltons (kDa). Band intensities were quantified by using ImageJ software. Resulting values are depicted as area above background (Supplementary Table S5).

**FIGURE 5:**
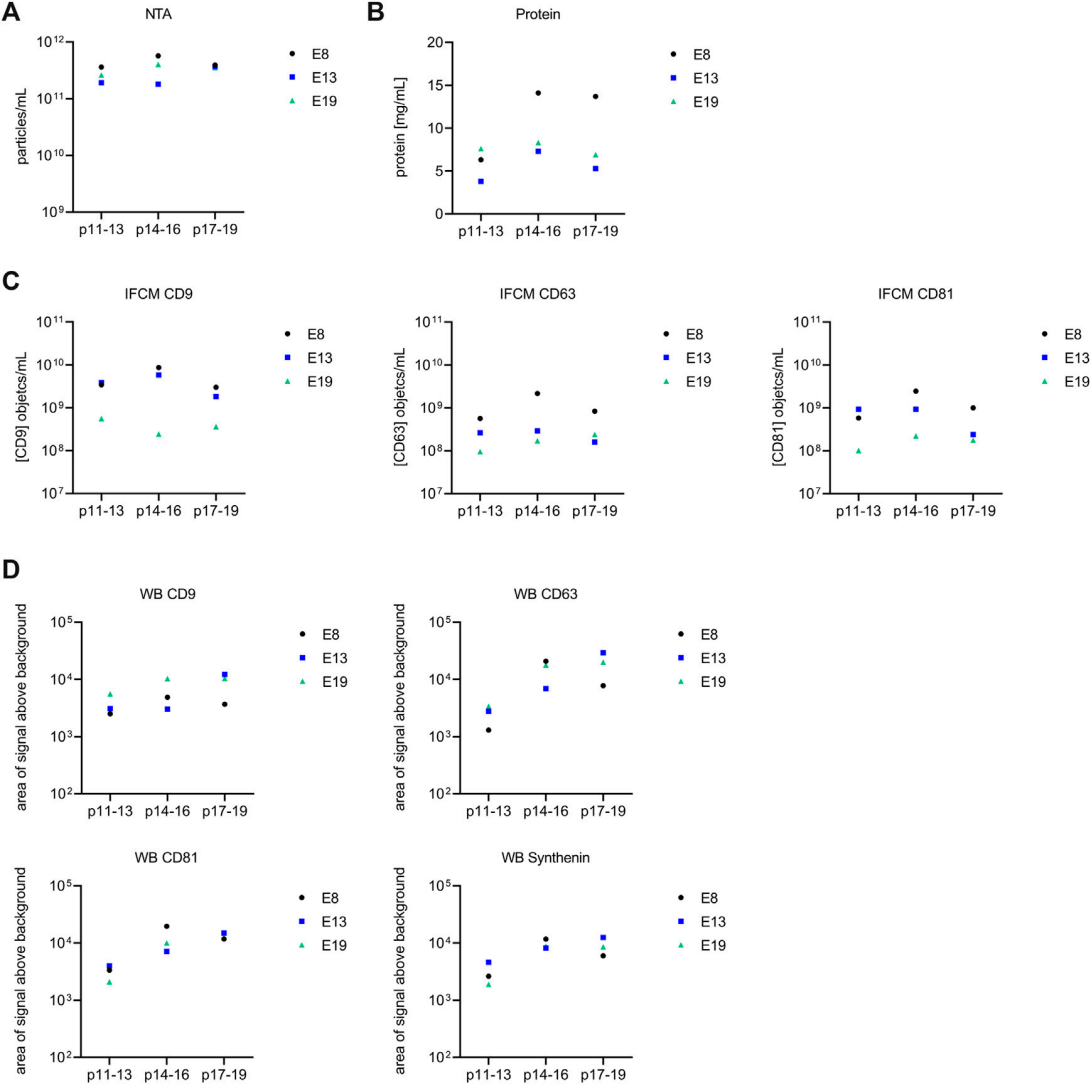
Comparative analysis of extracellular vesicle (EV) preparations from E8, E13, and E19. Each diagram provides a visual representation of the data (E8: black, E13: blue and E19: green). Values for each of the parameters are depicted in Supplementary Table S5. **(A)** Nanoparticle Tracking Analysis (NTA) results illustrating the particle concentration (particles/mL). **(B)** Protein content (mg/mL) of respective EV preparations. **(C)** Imaging flow cytometry (IFCM) results showing the concentration of objects/mL for CD9, CD63, and CD81. **(D)** Concentration of CD9, CD63, CD81 and Synthenin Western blot bands. Upon subjecting variations in the parameters to the different MSC induction protocols, no significant disparities were identified with the applied Mann-Whitney tests.

Samples of all given EV preparations were also characterized by Western blot, which was sequentially conducted for CD81, CD9 and CD63 on one blot and for Syntenin on a second blot (Figures 4A, B). Band intensities were quantified by using ImageJ software. Obtained data are provided in Supplementary Table S5 and are depicted in Figure 5B. In regards of CD81, considerable variations in band intensities were observed across the samples. The E8 p14-16 sample contained markedly higher CD81 levels than the other samples, the E13 p17-19 and E19 p17-19 samples also contained high CD81 concentrations. In contrast, E19 p11-13 and E13 p11-13 samples revealed bands with the lowest recorded intensities for CD81. For CD9, the E13 p17-19 and E19 p14-16 samples exhibited the highest band intensities, indicating a relatively elevated level of CD9 in these samples. Conversely, the E8 p11-13 and E13 p14-16 samples displayed the lowest band intensity. Anti-CD63 staining revealed that the highest CD63 concentration was found in the E13 p17-19 sample, pointing towards a robust expression level of CD63 in this sample. The bands with lowest intensities were obtained from E8 p11-13 and E13 p11-13 samples. Regarding Syntenin, E8 p14-16 and E13 p17-19 sample revealed the highest expression of Syntenin. Conversely, the lowest band intensity for Syntenin was recovered in the E19 p11-13 sample. Of note, upon applying Mann-Whitney tests for the recorded EV preparation parameters, no statistically significant impact of the MSC induction protocol was identified (E8 compared to E13 and E19).

**FIGURE 6:**
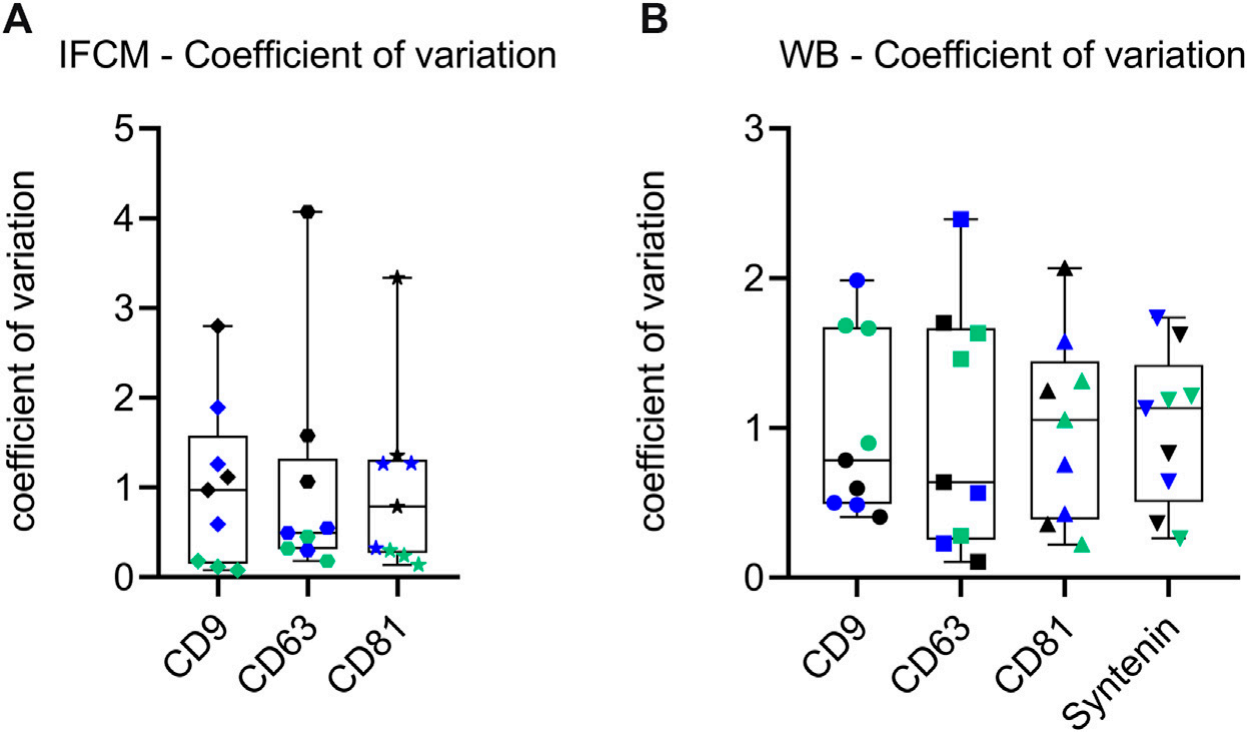
Analysis of sample coefficient of variation of recorded data reveal qualitative differences among the different iMSC-EV preparations. **(A)** Whisker dot plot representation of the sample coefficient of variation for Western blot band intensity data. **(B)** Whisker dot plot showcasing the sample coefficient of variation for imaging flow cytometry (IFCM) data. Individual data points are represented by dots, while whiskers delineate the span of variation. Each dot signifies an individual data point (E8: black, E13: blue and E19: green), with the whiskers indicating the range of variation. The collective representation underscores the spread and central tendencies of coefficients across both methodologies. The Brown-Forsythe test was conducted to assess the quality of variances in EV populations. The test yielded significant *p*-values of 0.004 for IFCM data and 0.035 for Western blot data, indicating quality differences in EV populations across different passages.

Even though the obtained metric data confirm that all iMSC-EV preparations fulfil given *bona fide* criteria for EVs (Thery et al., 2018), all metric analyses (Figures 5A–D) reveal variations among the individual iMSC-EV preparations. To investigate whether these variations are caused by handling issues, which would result in comparable variations among the different datasets, or rather by qualitative differences, i.e., different EV population compositions in the processed CM, the coefficients of variation for the IFCM and the Western blot data were determined. To this end, we conducted the Brown-Forsythe test, a reliable method for determining the quality of variances. Obtained *p*-values where 0.004 for the IFCM and 0.035 for the Western blot data (Figure 6), pointing towards significant differences within the quality of obtained EV populations. Consequently, the EV compositions were not consistent within the CM and varied among the different passages. Since iMSCs were always seeded and raised in a standardized manner (same cell number, same basal medium, supplements of the same batches, same containers), the data imply that the given variations derived from differences in the cell quality of the different passages.

Some but not all iMSC-EV preparations contain potentials to modulate allogenic immune responses *in vitro*.

For the evaluation of the immunomodulatory potentials of the obtained iMSC-EV preparations, samples of all iMSC-EV preparations were tested in the multi-donor mixed lymphocyte reaction (mdMLR) assay in comparison to appropriate controls. Here, upon pooling mononuclear cells from 12 different donors, strong allogenic immune responses are induced resulting in the activation of CD4 and CD8 T cells that can be recognized by their cell surface expression of CD25 and CD54. As reported in detail previously, the content of activated CD4 as well as of activated CD8 cells can be suppressed partially by immunomodulatory competent MSC-EV preparations (Bremer et al., 2023; Nardi Bauer et al., 2023).

**FIGURE 7:**
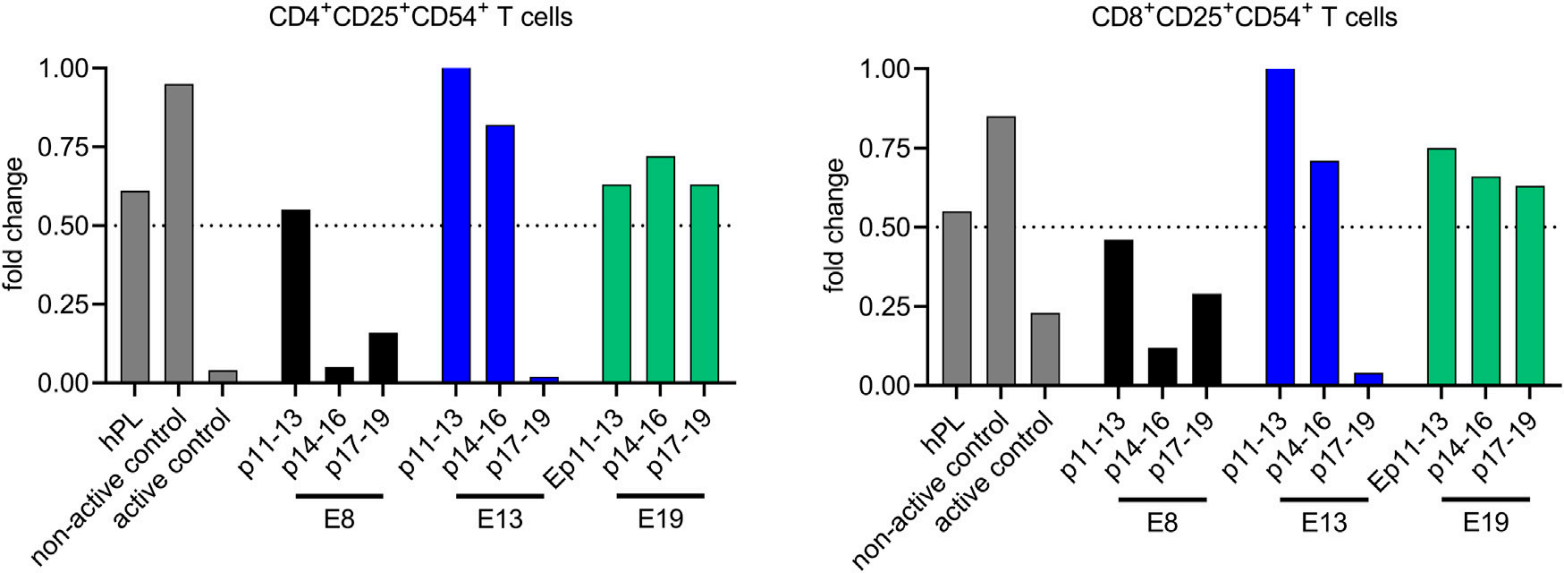
iMSC-EV preparations vary in their capability to suppress allogeneically activated CD4 and CD8 T cells in vitro. The immunomodulatory abilities of different iMSC-EV preparations (E8: black, E13: blue and E19: green) were assessed and compared to appropriate controls, i.e., in the absence of EVs or in the presence of active/non-active BM-MSC-EV samples (non-active control, active control). Additionally, EVs prepared from fresh, hPL-supplemented culture media were included as an extra control measure (hPL). The compositions of CD4 and CD8 T cells were scrutinized for the expression of the activation markers CD25 and CD54, markers indicative of T cell activation. The results are portrayed as ratios, representing the fold change of activated T cell content in EV-treated mdMLRs compared to EV-untreated mdMLRs. Only, samples suppressing the amount of activated CD4 T cells to less than 50% are considered to be active. A Mann-Whitney test was conducted to assess differences that might have been caused be the different MSC induction protocols; the analysis revealed no statistically significant variations.

Examining the impact of the different iMSC-EV preparations on the content of the CD25^+^CD54^+^ CD4 and CD8 T cells, and as depicted in Figure 7, we observed inconsistent effects. While EVs prepared from CM of iMSCs obtained in E19 marginally reduced the content of both populations, hardly any impact was observed for the EVs prepared from p14-p16 and p17-p19 CM of E13 iMSCs. In contrast, the strongest suppressive activity was monitored for the EV preparations of p11-13 CM of the E13 iMSCs. Activities of variable extends were also monitored for the iMSC-EV preparations of all E9 EV preparations. Thus, even though iMSCs obtained in our experiments are capable to release *bona fide* MSC-EVs, the obtained EV preparations varied non-predictable in their monitored immunomodulatory activity (Bremer et al., 2023; Madel et al., 2023). Thus, both, the metric data as well as the data of the functional analyses reveal inconsistencies among the given iMSC-EV preparations but no statistical differences.

## Discussion

Searching for feasible and scalable MSC-EV production strategies, in this study, we explored the capability of GMP-compliantly produced iPSCs to generate *bona fide* MSCs, determined their expansion and EV secretion capabilities and evaluated the immunomodulatory functions of obtained EV preparations *in vitro*. Comparable as other studies approved for research grade iPSCs (Lian et al., 2010; Giuliani et al., 2011; Ramos et al., 2022), we confirmed that GMP-compliant iPSCs are competent to create *bona fide* MSCs that can be extensively expanded apparently without overcoming the Hayflick limit. Upon using two different media to initially raise iMSC, one hPL supplemented and one commercial medium, we observed differences in expression of CD105 on progeny cells over several passages. While early passages of iMSCs initially kept in hPL supplemented media hardly expressed detectable amounts of CD105 on their cell surfaces and started to express higher levels at later stages (E8 iMSCs), iMSCs initially kept in a commercial MSC medium and transferred to hPL containing media in passages 7 and 8, expressed CD105 on their cell surfaces already in higher amounts in early passages. These observations confirm that the media initially used to raise iMSCs exert relevant and long-lasting impacts on the biology of the initial iMSCs and their progeny. Regarding the EV secretion performance of the obtained and expanded iMSCs, analyses of the obtained iMSC-EV preparations demonstrated that they significantly varied in their metric parameters and also differed regarding their immunomodulatory properties. Thus, despite approving that GMP-compliantly produced iPSCs are potent to develop into iMSCs that in principle can release immunomodulatory active EVs, the applied strategy fails to demonstrate consistency in the composition and function of obtained iMSC-EV products. Thus, for now, the chosen strategy does not overcome the limitations of classical MSC-EV production strategies that we addressed in our recent studies in which we used primary, BM-derived MSCs for the EV production:

As already mentioned in the introduction, we have observed that EV preparations being manufactured from CM of primary BM-derived MSCs frequently differ in their *in vitro* studied immunomodulatory capabilities as well as their therapeutic capabilities that have been studied in different animal models (Wang et al., 2020; Van Hoecke et al., 2021; Bremer et al., 2023; Madel et al., 2023; Nardi Bauer et al., 2023). In addition to donor-to-donor variations, we observe batch-to-batch variations with some MSC stocks having a higher and others a lower likeliness for the production of potent EV preparations (Madel et al., 2023; Nardi Bauer et al., 2023). Thus, such variations depend on the MSC stocks being used and assumedly on stochastic events. MSC propagations from primary cells are regularly polyclonal and have been reported to undergo clonal selection procedures (Selich et al., 2016). Apparently and very likely in donor specific manners, MSC stocks contain variable proportions of MSCs promoting or suppressing immunomodulatory activities of resulting EV preparations. Assumedly, the initial proportion of such MSC subtypes, combined with stochastic events during clonal selection procedures are decisive for the immunomodulatory *in vitro* capability of resulting MSC-EV products. Thus, with a certain probability, MSC stocks with a high prevalence for MSC-EV products with immunomodulatory capabilities can also result in products lacking these capabilities and *vice versa* (Nardi Bauer et al., 2023). In this context it is worth mentioning that phenotypic and functional heterogeneity of MSCs has been reported for more than 2 decades now (Phinney et al., 1999; Vogel et al., 2003; Phinney, 2012; Radtke et al., 2016). However, more intensive discussions about the impact of MSC heterogeneities, their origin and underlying culture conditions on their therapeutic potentials are just emerging (Dunn et al., 2021; Galipeau et al., 2021; Krampera and Le Blanc, 2021).

There are various considerations for the origin of such heterogeneities. On the one hand, the original tissues used as MSC source may contain different MSC progenitor types, as they have been discovered in hematopoietic stem cells niches (Wei and Frenette, 2018), that contribute in varying ratios to obtained MSC stocks. On the other hand, as it regularly takes several days before MSC progenitors start to proliferate, some epigenetic reorganisation may be required that hypothetically can contain some stochastic events sustainably affecting the biology of its MSC progeny. Even though, we do not have any evidence highlighting the one or the other explanation, for the origin of the MSCs’ intra donor heterogeneities, we consider that such heterogeneities occur in all polyclonal MSC populations, even if they are derived from clonally derived iPSCs. Indeed, in a previous study in which we have raised iMSCs from research grade iPSC, we observed varying immunomodulatory potentials of independently manufactured iMCS-EV preparations as well (Ramos et al., 2022). Remarkably, also in this study, iMSCs became senescent and were not expandable beyond passage 20 (Ramos et al., 2022). Thus, overall, even though larger product batches might become feasible with iMSC as starting source, to our best understanding they suffer from the same heterogeneity-based limitations than primary MSCs. Such limitations should massively challenge the setting up of robust, standardized and scalable EV production processes.

As highlighted in this study, MSC media severally can affect the biological properties of iMSCs. To this end it might be possible that the likeliness for potent MSC-EV products can be increased by optimising upstream production processes especially by the usage of an optimized MSC medium. However, we also should consider experiences from the therapeutic MSC field. Here, due to suboptimal MSC expansion conditions, a first phase III clinical trial in which adult steroid refractory aGvHD patients were treated with an MSC product failed to show efficacy (Galipeau, 2013; Kebriaei et al., 2020). After optimizing the MSC expansion, a second phase III clinical trial performed on paediatric aGvHD patients showed efficacy of the optimized MSC product (Kurtzberg et al., 2020a; Kurtzberg et al., 2020b). Still, the FDA repetitively did not provide the market authorisation for the product, for the second time in August 2023 (bit.ly/3OM2How). Without being aware for the reason of the second rejection, the argument for the first rejection in 2020 was that coupled to the insufficient expansion capability of primary MSCs, the MSC product has to be manufactured from varying donors. Expectably occurring impacts of donor-to-donor variations on the potency of the resulting drugs, were according to the FDA’s understanding not convincingly and sufficiently addressed (https://www.fda.gov/media/140988/download).

Aiming to avoid heterogeneity issues as much as possible and to also overcome MSC-aging related limitations, we thus have searched for a feasible strategy. To this end, we decided to immortalize MSCs and try to expanded them at the clonal level. Indeed, as described recently, we have successfully established this method and have expanded the first monoclonal cell lines for fare more than 100 population doublings without observing any indication of senescence, thus, clearly overcoming the Hayflick’s limit (Labusek et al., 2023). Notably, being expanded in appropriate media, some of these clonal immortalized MSC (ciMSC) lines repetitively allowed the preparation of EV products with confirmed *in vitro* immunomodulatory properties, while EV products of other ciMSC lines for now always failed to show such activities. Furthermore, we have confirmed the therapeutic potential of EV preparations obtained from a first ciMSC line *in vivo*. Combined to our specific interest in establishing novel treatment strategies for perinatal brain injuries (Jensen, 2023), ciMSC-EVs were administered to a murine model of neonatal hypoxia induced encephalopathy. The ciMSC-EV application significantly suppressed the disease symptoms at various cellular levels implying a multimodal mode of action that can hardly be mimicked by monomolecular compounds (Labusek et al., 2023). Thus, even though we cannot exclude that appropriate production strategies might overcome some of the discussed heterogeneity issues, we consider future MSC-EV therapeutics will derive from monoclonal MSC lines. In this context, it will be interesting to learn whether the original MSC source of such cell lines affects the therapeutic potency of resulting EV products and whether disease-specific preferred sources will be identified. Certainly, coupled to the confirmation that GMP-compliant iPSCs contain the potential to generate iMSCs that under certain conditions can release immunomodulatory active EVs, iPSCs remain an interesting source for the origin of MSCs for future MSC-EV production strategies.

## Data Availability

The original contributions presented in the study are included in the article/Supplementary Material, further inquiries can be directed to the corresponding author.
