# Supplementary material for: EV products obtained from iPSC-derived MSCs show batch-to-batch variations in their ability to modulate allogeneic immune responses in vitro
Source: Front Cell Dev Biol. 2023 Oct 30;11:1282860. doi: 10.3389/fcell.2023.1282860 (PMC10642442; doi:10.3389/fcell.2023.1282860)
Supplement: Supplementary file 1 [file Table1.DOCX]

***Suppl.-Table 1:*** *Applied fluorescence conjugated antibodies for cell characterization.*

| **Antigen** | **Conjugate** | **Host/isotype** | **Clone** | **Supplier** |
| --- | --- | --- | --- | --- |
| **Human CD14** | PO | Mouse IgG_1_ | MEM-15 | EXBIO |
| **Human CD31** | PE | Mouse IgG_1_ | 1F11 | Beckman Coulter |
| **Human CD34** | APC 750 | Mouse IgG_1_ | 581 | Beckman Coulter |
| **Human CD44** | APC | Mouse IgG2b, kappa | G44-26 | BD Biosciences |
| **Human CD45** | BV 785 | Mouse IgG_1_, kappa | HI30 | BioLegend |
| **Human CD73** | FITC | Mouse IgG_1_, kappa | AD2 | BD Biosciences |
| **Human CD90** | BV 605 | Mouse IgG_1_, kappa | 5E10 | BioLegend |
| **Human CD105** | BV 421 | Mouse IgG_1_, kappa | 43A3 | BioLegend |

APC: Allophycocyanin, FITC: Fluorescein isothiocyanate, BV: Brilliant Violet, PO: Pacific Orange; PE = Phycoerythrin

***Suppl.-Table 2****: This table summarises the compositions of the osteogenic and adipogenic induction media as well as of the adipogenic maintenance medium used in the experiment. The checkmarks (✔) indicate the presence of a specific component in the given medium, whereas the crosses (X) reflect its absence. hPL, Heparin, NaPy, PenStrep, Dexamethason, ß-Glycerolphosphat, Ascorbinsäure, Indomethacin, Insulin, and IBMX are the components of the media, DMEM high glucose is added to achieve the desired final volume.*

| **Medium** | **Osteogenic Differentiation Medium** | **Adipogenic Induction Medium** | **Adipogenic Maintenance Medium** |
| --- | --- | --- | --- |
| **hPL (10%)**  **(self-made)** | ✔ | ✔ | ✔ |
| **Heparin (0.1%)**  **(Ratiopharm)** | ✔ | ✔ | ✔ |
| **NaPy (1x)**  **(Sigma-Aldrich)** | ✔ | ✔ | ✔ |
| **PenStrep (1%)**  **(Gibco)** | ✔ | ✔ | ✔ |
| **Dexamethason**  **(100 μM)**  **(Sigma-Aldrich)** | ✔ | ✔ | ✔ |
| **ß-Glycerolphosphat**  **(1 M)**  **(Sigma-Aldrich)** | ✔ | X | X |
| **Ascorbinsäure**  **(300 mM)**  **(Sigma-Aldrich)** | ✔ | X | X |
| **Indomethacin**  **(200 mM)**  **(Sigma-Aldrich)** | X | ✔ | ✔ |
| **Insulin**  **(10 mg/ml)**  **(Sigma-Aldrich)** | X | X | ✔ |
| **IBMX**  **(50 mM)**  **(Sigma-Aldrich)** | X | X | ✔ |
| **DMEM high Glucose**  **(Gibco)** | ✔ | ✔ | ✔ |

***Suppl.-Table 3:*** *Applied fluorescence conjugated antibodies for the characterisation of obtained EV preparations*

| **Antigen** | **Conjugate** | **Host/isotype** | **Clone** | **Supplier** |
| --- | --- | --- | --- | --- |
| **Human CD9** | PE | Mouse, IgG1 | MEM-61 | EXBIO |
| **Human CD63** | APC | Mouse, IgG1 | MEM-259 | EXBIO |
| **Human CD81** | FITC | Mouse, IgG2a | JS64 | Beckman-Coulter |

***Suppl.-Table 4:*** *Applied fluorescence conjugated antibodies applied for the characterisation of cells harvested from the multi-donor mixed lymphocyte reaction assay.*

| **Antigen** | **Conjugate** | **Host/isotype** | **Clone** | **Supplier** |
| --- | --- | --- | --- | --- |
| **CD4** | BV785 | Mouse, IgG1 | RPA-T4 | BioLegend |
| **CD8** | BV650 | Mouse, IgG1 | SK-1 | BioLegend |
| **CD25** | PE | Mouse, IgG1 | BC96 | eBioscience |
| **CD54** | AF700 | Mouse, IgG2a | 1H4 | Exbio |

APC: Allophycocyanin, FITC: Fluorescein isothiocyanate, BV: Brilliant Violet, PO: Pacific Orange; PE = Phycoerythrin


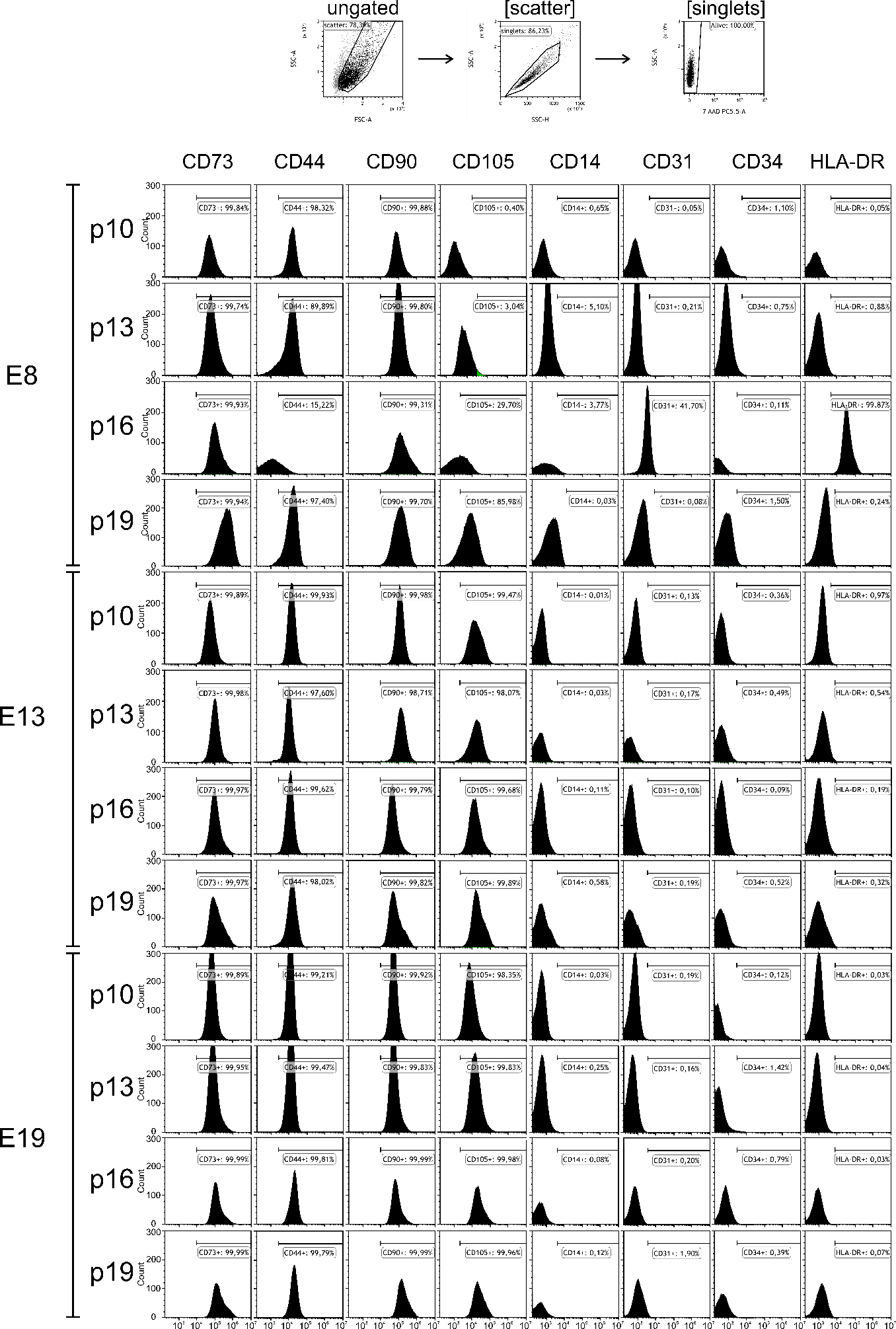


***Suppl.-Figure 1: Gating strategy and histogram plots for the cell surface phenotyping applied for the characterisation of iMSCs.***


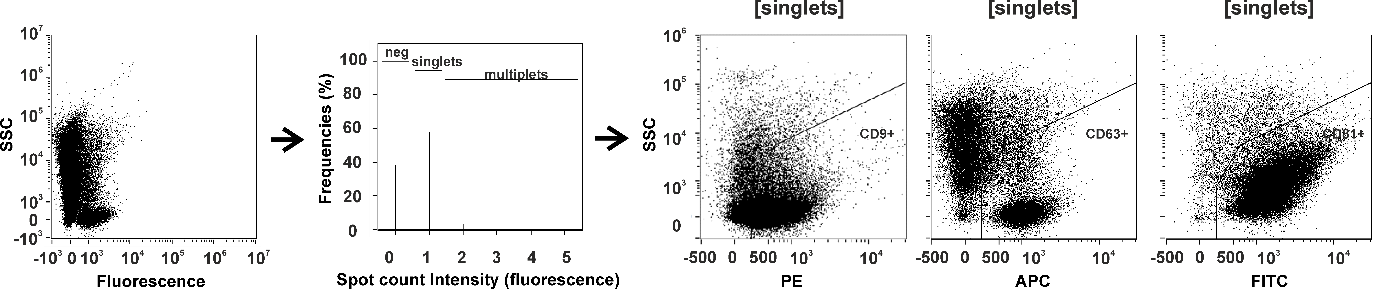


***Suppl.-Figure 2: Gating Strategy applied for the analsis of recorded imaging flow cytometry data of anti-CD9, anti-CD63 and anti-CD81 antibody stained iMSC-EV preparations.*** *The analysis begins with the total event population (far left plot), from which only singlet events are selected (second plot from left) for further analyses. These singlets are subsequently analysed for their fluorescence signals following staining with fluorescent antibodies. Recorded fluorescence data of the gated objects are ploated agains their side scatter (SSC) intensities. Of note CD9^+^ events are PE-labeled, CD63^+^ objects are APC-labeled and CD81^+^ objects are FITC-labeled.*

Suppl.-Table 5: Metric characteristics of given iMSC-EV preparations. Obtained data from nanoparticle tracking analysis (NTA) provided as particle concentration per ml (Particle/ml), the protein concentration (mg/ml), and the concentrations of CD9+, CD63+ and CD81+ objects per ml as determined by imaging flow cytometry (IFCM). All data were collected from the three independent experiments, E8, E13, and E19 and of all three independent EV preparations per experiment (p11-13, p14-16, p17-19).

|  | **NTA** | **NTA** | **Protein** | **IFCM CD9** | **IFCM CD63** | **IFCM CD81** | **WB**  **CD9** | **WB**  **CD63** | **WB**  **CD81** | **WB**  **Synthenin** |
| --- | --- | --- | --- | --- | --- | --- | --- | --- | --- | --- |
|  | [Particle/ml] | Size [nm] | [mg/ml] | [Objects/ml] | | | [Area above background] | | | |
| **E8 p11-13** | 3.6x10¹¹ | 118.9 | 6.3 | 3.4x10⁹ | 5.8x10⁸ | 5.8x10⁸ | 2.5x10^3^ | 1.3x10^3^ | 3.4x10^3^ | 2.6x10^3^ |
| **E8 p14-16** | 5.7x10¹¹ | 120.3 | 14.1 | 8.6x10⁹ | 2.3x10⁹ | 2.5x10⁹ | 4.9x10^3^ | 2.1x10^4^ | 2.0x10^4^ | 1.2x10^4^ |
| **E8 p17-19** | 3.9x10¹¹ | 114.7 | 13.7 | 3.0x10⁹ | 8.4x10⁸ | 1.0x10⁹ | 3.7x10^3^ | 7.8x10^3^ | 1.2x10^4^ | 6.0x10^3^ |
| **E13 p11-13** | 1.9x10¹¹ | 123.6 | 3.8 | 3.9x10⁹ | 2.6x10⁸ | 9.3x10⁸ | 3.1x10^3^ | 2.8x10^3^ | 4.0x10^3^ | 4.6x10^3^ |
| **E13 p14-16** | 1.8x10¹¹ | 119.8 | 7.3 | 5.8x10⁹ | 2.9x10⁸ | 9.3x10⁸ | 3.0x10^3^ | 6.9x10^3^ | 7.1x10^3^ | 8.2x10^3^ |
| **E13 p17-19** | 3.7x10¹¹ | 118.9 | 5.3 | 1.8x10⁹ | 1.6x10⁸ | 2.4x10⁸ | 1.2x10^4^ | 2.9x10^4^ | 1.5x10^4^ | 1.3x10^4^ |
| **E19 p11-13** | 2.6x10¹¹ | 112.1 | 7.6 | 5.6x10⁸ | 9.5x10⁷ | 1.0x10⁸ | 3.0x10^3^ | 3.4x10^3^ | 2.1x10^3^ | 1.9x10^3^ |
| **E19 p14-16** | 4.0x10¹¹ | 110.3 | 8.3 | 2.4x10⁸ | 1.7x10⁸ | 2.2x10⁸ | 1.0x10^4^ | 1.8x10^4^ | 1.0x10^4^ | 8.8x10^3^ |
| **E19 p17-19** | 3.5x10¹¹ | 109.8 | 6.9 | 3.6x10⁸ | 2.4x10⁸ | 1.6x10⁸ | 1.0x10^4^ | 2.0x10^4^ | 1.2x10^4^ | 8.9x10^3^ |
